# Pretreatment Effect of Folic Acid on 13-Cis-RA-Induced Cellular Damage of Developing Midfacial Processes in Cultured Rat Embryos

**DOI:** 10.2174/1874210601711010200

**Published:** 2017-03-31

**Authors:** Rungarun Kriangkrai, Suconta Chareonvit, Sachiko Iseki, Visaka Limwongse

**Affiliations:** 1 Department of Oral Biology, Faculty of Dentistry, Naresuan University, Phitsanulok, Thailand.; 2 Department of Anatomy, Faculty of Dentistry, Chulalongkorn University, Bangkok, Thailand; 3 Department of Molecular Craniofacial Embryology, Graduate School, Tokyo Medical and Dental University, Tokyo, Japan

**Keywords:** Apoptosis, Cell proliferation, Folic acid, Midfacial process, 13-Cis-RA, Rat embryo

## Abstract

**Objective:**

Excess treatment of 13-cis-RA (Accutane^®^) on pregnant women induces craniofacial malformation found in infants. However, the effect of folic acid on 13-cis-RA-induced cellular damages of developing midfacial processes is still unknown. The purpose of this study was to investigate the pretreatment effect of folic acid (FA) on 13-cis-RA-induced cellular damage in developing midfacial processes in rat embryos.

**Materials and Methods:**

The rat embryos at developing midfacial processes were performed by whole embryo culture *in vitro*, in the presence of 13-cis-RA (20 µM) with or without pre-treatment of FA (100 µM). The midfacial morphogenesis score, PCNA and TUNEL assay staining were evaluated for morphogenesis, cell proliferation and apoptosis of the midfacial processes, respectively.

**Results:**

The 13-cis-RA-treated embryos at 24h showed atrophy of midfacial processes with significantly decreased morphogenesis score and cell proliferation, and increased apoptotic cell death. In contrast, the embryos pre-treated with FA for 18h, followed by 13-cis-RA treatment for 24h (FA-RA) showed significantly greater morphogenesis score, increased cell proliferation and lower apoptotic cell death compared to those of the 13-cis-RA-treated embryos.

**Conclusion:**

The results suggest that FA reduced the teratogenic effects of 13-cis-RA on midfacial process tissue. Future investigations regarding the anti-teratogenic mechanism of FA on the prevention of damages in midface processes induced by 13-cis-RA on pregnant woman are warranted.

## INTRODUCTION

Human face develops during the 4^th^ and 10^th^ weeks of embryonic development. Starting at the end of week 4, five facial processes consisting primarily of neural crest-derived mesenchyme appear, namely the frontonasal process (FNP) constituting the upper border of stomodeum, a pair of maxillary processes (MxPs) lateral to the stomodeum and a pair of mandibular processes (MdPs) constituting the lower border of stomodeum. During the 5^th^ week, the thickening of surface ectoderm called nasal placodes can be found on both sides of FNP. The placodes invaginate to form nasal pits, while a part of FNP divides two medial nasal processes (MNPs) and two lateral nasal processes (LNPs). A series of facial processes and their mergences give rise to the different parts of the face. During the 7^th^ week, the MxPs continually grow and increase in size. The lower portions of MxPs merge with the MNPs to form upper lip, resulting in the loss of clefts between MNPs and MxPs. The upper part of MxPs merges with LNPs covering nasolacrimal duct to form upper cheeks and maxillae while the LNPs generate lateral parts and alae of nose. Moreover, the growth of MxPs can compress MNPs medially to merge at midline. The upper part of merged MNPs generates the dorsum and tip of nose while its lower part generates the intermaxillary segment. Intermaxillary segment subsequently forms the philtrum of the upper lip, the upper jaw component caring the four incisor teeth and the primary palate. The MdPs merge across the midline to form the lower lip, cheek and jaw. The palate is formed by the fusion of primary palate and palatal processes growing from the oral surfaces of the maxillary processes [1-3]. The union of midfacial processes including the MNPs, LNPs and MxPs yields an area between the upper lip and forehead, called the midface. Animal studies showed that the midface was started by the formation of recognized FNP and MxPs at embryonic day (E) 11.0 in rat embryos, comparable to E 9.0 in mouse embryos. Subsequently, FNP developed and bulged around the nasal pits forming the horseshoe-shaped lateral and medial nasal processes (LNPs and MNPs) at stage E 11.5 in rat embryos. At E 12.0 in rat embryos, MNPs and LNPs were formed properly. The mesenchyme of MxPs rapidly grew and merged with the MNPs to form the upper lip. Interruption in the growth and merging of midfacial processes during E11-12 in rat embryos, may lead to facial defects such as cleft lip. The cleft lip is a merging failure between the MxPs and MNPs, while the median cleft lip is a merging failure of the MNPs into midline [4, 5].

Retinoic acid (RA), an analogue of vitamin A, is commonly used in dermatology for the treatment of severe cystic acne, ichthyosis and keratinizing dermatoses [6]. Endogenous RA plays the key roles in a variety of biological processes and embryonic development such as immune function, reproduction [7-9] and maintenance of normal growth pattern [10]. In contrast, an excessive intake of RA during pregnancy results in birth defects termed “retinoic acid embryopathy” [11, 12]. Craniofacial region has been known as one of the most RA sensitive structures because mostly craniofacial mesenchyme is derived from cranial neural crest cells (CNCs) [13], which are primary target tissue for RA-induced damage [14]. The damages include exencephaly, hydrocephalus, cleft lip with or without cleft palate, and shortening of maxilla and mandible. The severity of malformation depends on the dose and embryonic stage at the time of drug administration [11, 15-17]. The 13-cis-RA or Acccutane^®^ is a retinoic acid derivative that poses several benefits such as treating severe cystic acne [18]. However, the clinical observations strongly have suggested that the 13-cis-RA is a human teratogen. Human pregnancies exposed to the 13-cis-RA had a high incidence of ear malformation and cleft palate in infants [11]. Malformations induced by 13-cis-RA in human closely resembles to those occurring in laboratory animals [5, 19]. The previous studies reported that 13-cis-RA was clearly toxic to E10 mouse embryos. When the embryos exposed to 10 µM 13-cis-RA for 24 in the culture, the mesenchymal cell death showing pyknotic nuclei was found beneath the epithelium of midfacial processes [20]. A high percentage of median cleft lip indicating the merging failure of MNPs was observed in the high dose exposure (20 µM) of 13-cis-RA for 48 h. Moreover, 13-cis-RA at 20 µM also inhibited the frontonasal mesenchyme proliferation in primary culture [5]. Collectively, these results suggested that 13-cis-RA at concentrations of 10-20 µM was clearly toxic to midfacial process development and CNCs-derived mesenchyme populated in midfacial processes was the target of RA-induced damage.

Folate (pteroiloglutamic acid) or folic acid (FA) (synthetic form) is one of the most vital substrates for cell metabolism. Folate participates in (1) DNA biosynthesis (guanine, adenine and thymine) essential for cell division, and (2) in embryogenesis (Fig. **6**) by serving as a cofactor and co-substrate for the synthesis of methionine and S-adenosylmethionine (SAM) necessary for methylation reactions in gene expression process [21]. An *In vitro* study showed that FA was beneficial for cranial and cardiac neural crest cell proliferation and differentiation. FA promoted neuroepithelial cell outgrowth and increased neural crest cell differentiation [22]. Recently, *in vivo* and *in vitro* studies-demonstrated that FA had an inhibitory effect on apoptosis induced by drugs or teratogens in neural crest cell derivative tissues [23-25]. These studies have emphasized the role of FA in cell proliferation and gene expression which are highly important during early embryogenesis. Indeed, FA is crucial for the survival of CNC derivative tissues, such as midfacial processes, which are the target tissue damaged by excess RA. To date, no one has previously reported the roles of FA on RA induced tissue damage of the midfacial processes. Therefore, the objective of this study was to determine whether pre-treatment of FA had protective effects on 13-cis-RA-induced cellular damage of developing midfacial processes in cultured rat embryos.

## MATERIALS AND METHODS

### Animals

Sprague-dawley (SD) rat embryos were used in this study. Vaginal plug appearance was designated as day 0 of embryogenesis (=E0). All experiments were performed in accordance with protocols certified by the Institutional Animal Care and Use Committee of Naresuan University, Phitsanulok, Thailand.

### Chemicals

13-cis-retinoic acid (13-cis-RA), Folic acid (FA), and DMSO were purchased from Sigma Chemical Company (St. Louis, MO, USA). 13-cis-RA was dissolved in DMSO and diluted to a final concentration of 20 µM in the culture medium. DMSO concentration was 0.05% and this concentration was added into the control culture as vehicle control. FA was dissolved and diluted to a final concentration of 100 µM in culture medium.

### Whole Embryo Culture

**Fig. (1):**
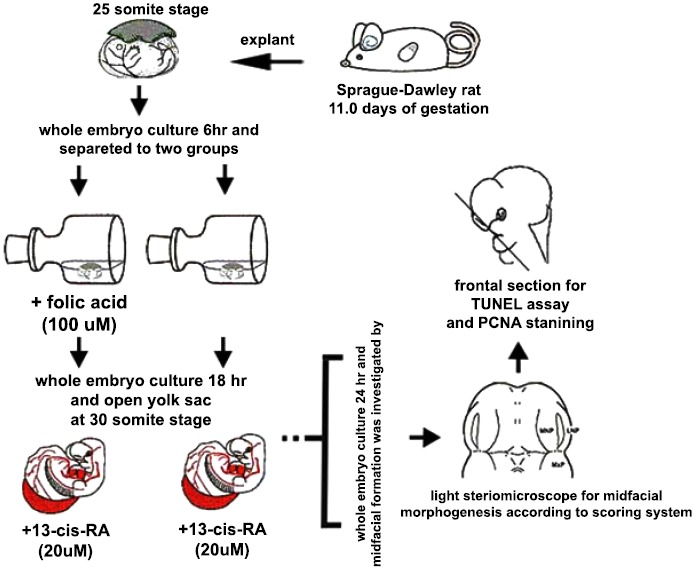
Schematic illustration of whole embryo culture and treatments. E11.0 SD-rat embryos were explanted and cultured for 48 h. They were separated into two experimental groups: (1) pre-treatment of 100 µM folic acid for 18h, followed by 20 µM 13-cis-RA for 24h (FA-RA), (2) treatment of 20 µM 13-cis-RA alone for 24h (RA). After the culture, midfacial morphogenesis was investigated with morphologic scoring system by light stereomicroscope. The midfacial processes of the embryos were also frontally sectioned for TUNEL assay, and PCNA staining.

The E11.0 SD-rat embryos displaying 25 somite stage, the initial stage of FNP and MxP formation, were selected for the whole embryo culture (Fig. **1**) [4]. The embryos were explanted and individually cultured in a bottle containing 2 ml of rat serum with 2 mg/ml glucose, in continuous gas condition (60% O_2_ and 5% CO_2_ balanced with N_2_) for 6 h. Then, embryos were divided into two experimental treatment groups (n=18) as follows: (1) FA-RA: pre-treatment of 100 µM FA for 18 h, followed by 20 µM 13-cis-RA for 24 h (FA-RA treated embryos), (2) RA: 20 µM 13-cis-RA alone for 24 h (13-cis-RA treated embryos). The 13-cis-RA treatment was applied to the embryonic culture at the 30-somite stage after the yolk sac was opened. The formation of nasal pit surrounded by the swelling of MNPs and LNPs was observed, resembling to E11.5 of rat embryogenesis *in vivo*. Subsequently, the gas supply of the culture condition was changed to 90% O_2_ and 5% CO_2_. At the time of each experiment, the 0.05% DMSO-treated embryos (DMSO, n=19) were performed as a control of the 13-cis-RA vehicle, and 100 µM FA-treated embryos (FA, n=18) were also performed as a control of FA treatment alone. After 48 h, all cultured embryos showed their embryogenesis resembling to E12 of rat embryogenesis *in vivo* as the midfacial processes (MNPs, LNPs and MxPs) were formed and started to merge with each other. The MxPs pushed MNPs to join and merge medially at the midline. The midfacial morphogenesis of all embryos was evaluated under light stereomicroscope (40X, SZX16, Olympus, Japan) according to the scoring system (Table **1**, adapted from [26]). To represent the morphogenesis of midfacial processes of each group, the embryos were selected and processed through scanning electron microscopy (SEM). By limitation of culture technique, the formation and fusion of midfacial processes were evaluated only on the left side of embryos due to the interference of placenta on the right side after the yolk sac was opened. The midfacial processes were also frontally sectioned for TUNEL assay, and PCNA staining.

### Scanning Electron Microscopy (SEM)

The heads of the control and treated embryos were dissected and fixed in Bouin’s solution. The samples were dehydrated through a graded series of ethanol and transferred to a critical point dryer with transitional fluid CO_2_ (CPD; Polaron 7501, East Sussex, UK). Samples were mounted onto aluminum pin stubs, coated with a thin layer of gold using a sputter coater (Spi supplies; 12155-AX, Pennsylvania, US) and examined under a scanning electron microscope (Zeiss; LEO 1455VP, New Jersey, US).

### TUNEL Assay and PCNA Staining

The heads of the control and treated embryos (n=6) were dissected and fixed in 4% paraformaldehyde (PFA) dissolved in PBS at 4 ºC, followed by dehydration through a graded series of ethanol before being embedded in paraffin. Specimens were sectioned (5 µm) for a TUNEL assay to detect apoptotic cells (the DeadEnd Colorimetric TUNEL system, Promega Co., Madison, WI) and exposed to PCNA staining to detect cell proliferation (PCNA staining kit, Invitrogen Corporation, Flynn Rd, Camarillo) according to the manufacturer’s instruction. The sections were counterstained with hematoxylin. The TUNEL-positive cells or PCNA positive cells shown in brown color were counted in a fixed area (150 µm in width x 400 µm in height). The ratio of positive stained cells over total cell number (%) was calculated in each sample.

### Statistical Analysis

All statistical analyses were performed with a standard statistical package (SPSS Inc., Chicago, IL, USA). Data were analyzed using Kruskal-Wallis test and presented as means ± standard error of the mean (SEM). The Mann-Whitney test was used to evaluate significant differences among groups. The level of significance was *p* < 0.05.

## RESULTS

### Pretreatment of FA Reduced the Malformation of Midfacial Processes Induced by 13-cis-RA

**Fig. (2):**
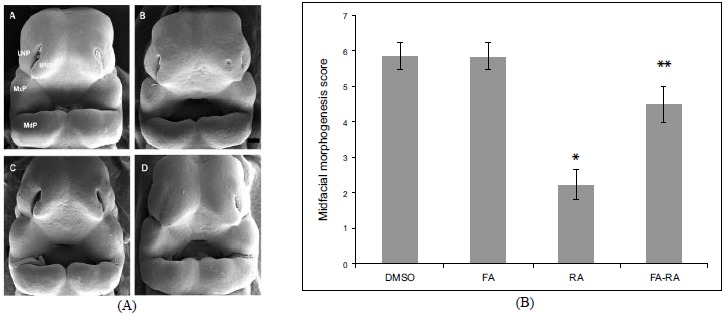
Scanning electron micrographs of rat embryos treated with 0.05% DMSO (A), 20 µM 13-cis-RA (B), 100 µM FA (C) and pre-treatment of 100 µM FA, followed by 20 µM 13-cis-RA (FA-RA) (D) *in vitro*. (A) and (C) showed normal midfacial formation (score=6). (B) showed teratogenic effect of 13-cis-RA induced atrophic of midfacial processes (score=2) presenting the lowest score of developing midfacial processes. (D) showed the protective effects of FA on RA induced malformation resulting in increased midfacial process score (score=5), Scale bar = 200 µm. (b): By scoring system assessment, there were no significant differences in midfacial morphogenesis score between DMSO-treated (n=19) and FA-treated embryo (n=18). The midfacial morphgenesis score of 13-cis-RA treated group significantly decreased compared to that of the DMSO-treated group. The FA-RA treated embryos showed significantly increased in midfacial morphgenesis scores compared to 13-cis-RA-treated group (RA). *p < 0.05 vs. DMSO and FA; **p < 0.05 vs. RA.

There was no significant difference in the midfacial morphogenesis score between the FA-treated group (5.800 ± 0.410) and the DMSO-treated group (5.842 ± 0.374) (Fig. **2**). The midfacial morphogenesis score of the 13-cis-RA-treated group (2.222 ± 0.427) was significantly less than that of the DMSO-treated group. Notably, the midfacial morphogenesis score of the FA-RA-treated group (4.5 ± 0.514) was significantly greater than that of the 13-cis-RA-treated group (Fig. **2**).

### Pretreatment of FA Protected Against Apoptosis and Contributed to Cell Proliferation in the Midfacial Processes of 13-cis-RA-Treated Embryos

**Fig. (3):**
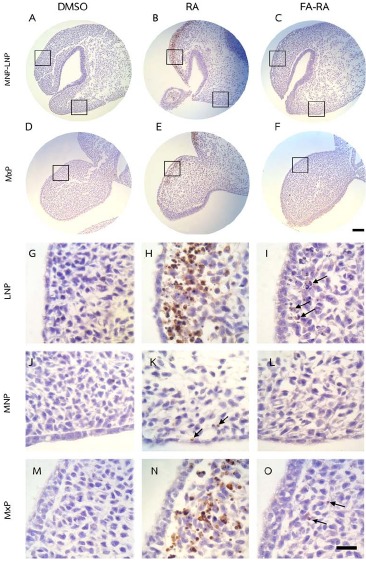
TUNEL assay of embryos treated with DMSO, 13-cis-RA (RA) and a combined folic acid and 13-cis-RA (FA-RA). The 13-cis-RA treated embryos showed numerous TUNEL-positive cells (brown staining) in the mesenchyme adjacent to the epithelium of dorsolateral part of LNP and MxP, (B, E, H, N) compared to those of the DMSO-treated embryos. A lower amount of positively stained cells found in 13-cis-RA treated embryos noted in the MNP (K, arrows). A dramatic reduction of TUNEL-positive cells was particularly found in the LNP and MxP of FA-RA-treated embryos (4 C, F and I, O arrows) compared to those observed in the 13-cis-RA treated embryos. G-O showed a higher magnification of square area in A-F. Scale bar= 100 µm for A-F and 20 µm for G-O.

**Fig. (4):**
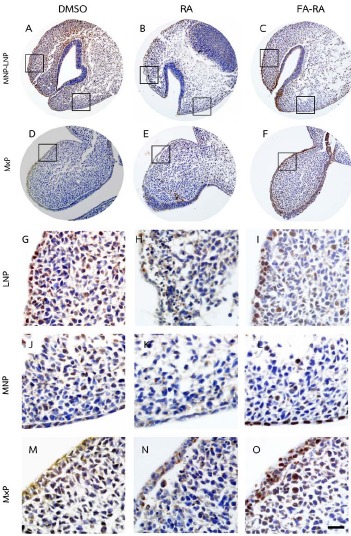
PNCA assay of embryos treated with DMSO, 13-cis-RA (RA) and a combined folic acid and 13-cis-RA (FA-RA). In DMSO-treated embryos, numerous PCNA positive cells (brown staining) were found in epithelium and mesenchyme of midfacial processes (A, D, G, J, M). The 13-cis-RA-treated embryos showed lower numbers of PCNA positive cells found in epithelium and mesenchyme of LNP, MNP and MxP (B, E, H, K, N), compared to those of the DMSO-treated embryos. The FA-RA-treated embryos showed an increased numbers of PCNA positive cells in epithelium and mesenchyme of LNP, MNP and MxP (C, F, I, L, O), compared to those of the RA-treated embryos. G-O showed a higher magnification of square area in A-F. Scale bar= 100 µm for A-F and 20 µm for G-O.

**Fig. (5):**
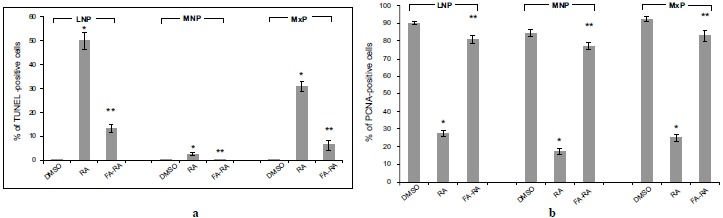
Quantitative assessment of TUNEL (a) and PNCA assay (b) representing the ratio of positive stained cells over total cell number (%). (a) TUNEL assay. The 13-cis-RA significantly induced apoptotic cell death (more TUNEL-positive cells) in LNP, MNP and MxP of 13-cis-RA treated embryos compared to DMSO treated embryos. A significant reduction of TUNEL-positive cells was observed in FA-RA-treated embryos compared to those of the 13-cis-RA-treated embryos. (b): PCNA staining. The 13-cis-RA treated embryos revealed significantly decreased cell proliferation (less PCNA-positive cells) in midfacial processes compared to that of DMSO-treated embryos. Midfacial processes of FA-RA treated embryos showed significantly higher an increased cell proliferation than that of 13-cis-RA -treated embryos. Note: *p < 0.05 vs. DMSO; **p < 0.05 vs. RA.

The DMSO-treated embryos showed low numbers of TUNEL-positive cells (brown staining) and high numbers of PCNA-positive cells (brown staining) in midfacial processes (Figs. **3** and **4A**, **D**, **G**, **J**, **M**). There was no significant difference in the TUNEL- and PCNA-positive cells in midfacial processes between DMSO- and FA-treated embryos (data not shown). As a result, the DMSO-treated embryos were used as a control for these experiments. 13-cis-RA showed the teratogenic effect of apoptosis induction. The effect was mostly found in mesenchymal cells compared to epithelial cells of the midfacial processes of treated embryos. The TUNEL-positive stained cells were significantly observed in the mesenchymal cells underneath the epithelium lining of LNP and MxP (Figs. **3B**, **E**, **H**, **N**) of the 13-cis-RA treated embryos compared to the DMSO-treated embryos (Fig. **5a**). Even though small numbers of TUNEL-positive stained cells of 13-cis-RA-treated embryos were observed in the MNP (Figs. **3B**, **K** arrows), they were significantly higher than those observed in DMSO-treated embryos (Fig. **5a**).

A comparison between the 13-cis-RA- and FA-RA-treated embryos indicated that FA-RA-treated embryos had significant reduction in the TUNEL-positive cells in the dorsolateral part of LNP, MxP and MNP (Figs. **3C**, **F**, **I**, **O** arrows, and **L**) compared to those observed in 13-cis-RA-treated embryos (Fig. **5a**). Conversely, PCNA staining, the 13-cis-RA-treated embryos showed significantly lower numbers of positive stained cells in the epithelium and mesenchyme of the midfacial processes than those observed in the DMSO- and FA-RA-treated embryos (*p* <0.05, Figs. (**4**, **5b**)).

## DISCUSSION

Rat embryos were initially cultured at E11.0 stage and the formation of FNP and MxPs were observed. After 24 h of the culture, FNP consequently divided into MNPs, LNPs in resemblance of their development at E11.5 of embryogenesis *in vivo*, showing the formation of midfacial processes, not yet merged into midline. At this stage, the embryos were exposed to 20 µM 13-cis-RA for 24h. The 20 µM 13-cis-RA hindered the formation of midfacial processes of rat embryos leading to the decreased morphogenesis score of 13-cis-RA-treated embryos. The atrophic formation of midfacial processes was found showing an improper growth and merging of the MNPs, LNPs and MxPs. Furthermore, when the embryos were exposed to 20 µM 13-cis-RA for a longer period (42h), severe atrophy of midfacial processes was observed resulting in the failure of their growth and mergence (data not shown). This was clearly supported by the limited cell proliferation and a larger number of apoptotic cells in the mesenchyme of 20 µM 13-cis-RA-treated embryos. The results of this study were similar to the previous reports in mouse embryos exposed to the 13-cis-RA. E10 mouse embryos were treated with 10 µM 13-cis-RA for 24h showing the mild atrophy of MNPs and LNPs, as well as a reduction in mesenchymal cell proliferation and an increased cell death in MNPs, LNPs, and MxPs. More serious defects such as a high incidence of median cleft lips were also observed when the embryos exposed to 20 µM for 48h [5, 20]. In our study, the TUNEL assay revealed high numbers of apoptotic cells in midfacial mesenchyme, especially in LNPs, of 13-cis-RA-treated embryos. Normally, RA signaling is necessary for epithelial-mesenchymal interaction supporting the FNP formation [27]. The endogenous RA is synthesized in FNP epithelium, and signals through to the receptor in neural crest cell derived-mesenchyme that populates in the FNP. This RA signaling drives *via* nuclear dimer of RAR and RXR, resulting in RARE activation and promotion of downstream gene expression such as *RARβ* in mesenchyme. The *RARβ* expression representing the RA signaling in the mesenchyme signals the epithelium to express the essential genes including *FGF8* and *SHH*. The expression of *FGF8* and *SHH* maintains the cell survival and cell proliferation of the mesenchyme essential for the FNP outgrowth [28, 29]. On the other hand, disruption of endogenous retinoid signaling in the developing FNP leads to the lack of *FGF8* and *SHH* expression that eventually causing increased programmed cell death and decreased cell proliferation in mesenchyme of FNP [28]. At the later stage when FNP developed into MNPs and LNPs, the *RARβ* expression representing RA signaling is locally found in LNPs. Correlation of retinoid signaling inhibition and the loss of *FGF8* expression has contributed to the specific increase in programmed cell death in the LNPs. This malformation is not observed in MNPs [29]. The results suggested that retinoid signaling is essential for LNPs formation. In accordance to our results, an excessive uptake of RA during midface formation negatively affected LNPs formation showing decreased cell proliferation and increased apoptosis, suggesting that either excess or shortage of RA signaling induces a similar abnormal phenotype found in the midfacial processes, especially in LNPs [28, 29]. However, the pathogenesis and the downstream of target genes inducing apoptosis in LNPs by excess RA are not well understood. To date, previous reports in mouse embryos showed that *RARβ* was normally expressed in developing LNPs, but was not expressed in MNPs and MxPs. Excessive uptake of RA in mouse embryos highly induced the *RARβ* expression and cell death in the mesenchyme of the midfacial processes. The high *RARβ* expression region induced by an excess uptake of RA included the area where the extreme cell death was observed. These results were mainly found in the LNPs and MxPs, suggesting that an uptake excess RA might have induced the malformation via the *RARβ* responsive signaling in specific mesenchyme of LNPs and MxPs [20, 30, 31]. An *in vitro* study showed that mesenchymal cells of LNP and MxP were highly responsive to RA showing an increased *RARβ* expression, while mesenchymal cells in the MNP were lowly responsive [30]. It has been suggested that the different characters of CNCs-derived mesenchyme among individual midfacial process were dependent on the difference of their neural crest origin [32]. As a consequence as shown in our study, excess RA mainly affected the mesenchyme of the LNPs and MxPs, but mildly affected the MNP formation. The LNPs are the lateral part of the nasal process which fuse with the MNPs and MxPs. During midfacial morphogenesis, the LNPs support the fusion of the MxPs to the MNPs and also promote the fusion of MNPs with the contralateral side. Therefore, the atrophic LNPs would impact the development of the other midfacial processes, *i.e*. MxPs and MNPs.

In addition, our data demonstrated that exogenous RA could inhibit the epithelial and mesenchymal cell proliferation in developing midface as shown by a low density of expressed PCNA in the midfacial processes of RA-treated samples (Figs. **4**, **5b**). RA greatly reduced epithelial cell proliferation, but poorly induced epithelial cell apoptosis, suggesting that the inhibition of cell proliferation is the main adverse effect of RA on epithelial cells of midfacial processes. However, the underlying mechanism of this action has not been elucidated. Our results suggested that the exogenous RA increases the RA signaling in the mesenchyme underneath the epithelium lining, resulting in an induced mesenchymal cell apoptosis. This process could, consequently, disrupt the signals from mesenchyme to epithelium, hence the interruption of genes expression maintenance such as *FGF8* and *SHH* in epithelium. These genes are necessary for cell survival and cell proliferation in the mesenchyme not only via paracrine function, but also by autocrine function in order to maintain epithelial cell proliferation of the midfacial processes [33-35]. Thus, their expressions in ectoderm have correlatively controlled the outgrowth pattern of midface and other craniofacial organs [36-38].

**Fig. (6):**
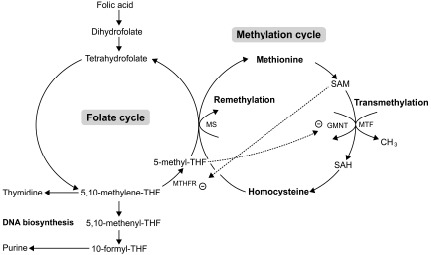
Schematic illustration showing the relationship between methylation cycle and folate metabolism. Folate metabolism is necessary for DNA biosynthesis and is required for remethylation process. SAM, a methyl group donor in transmethylation reactions, is produced from dietary methionine and participates in the remethylation of homocysteine in a vitamin B12-dependent reaction using the folate-dependent one-carbon pool as a methyl group source (5-methyl-THF). SAM/SAH is an index of transmethylation potential. SAM acts as an allosteric inhibitor of methylene-THF reductase (MTHFR), the enzyme catalyzed the irreversible reduction of 5, 10-methylene-THF to 5-methyl-THF. In turn, 5-methyl-THF is an inhibitor of GNMT, a key protein involved in the regulation of transmethylation by controlling the SAM/SAH ratio. THF= tetrahydrofolate, GNMT=N-methyltransferase, SAM= S-adenosyl methione, SAH=S-adenosyl homocysteine, MS= Methionie synthase (Vitamin B12), MTF= Methyltransferase.

On the other hand, FA had protective effects on 13-cis-RA-induced cellular defects of midfacial processes by reducing apoptotic cell death (Fig. **5a**), and enhancing cell proliferation (Fig. **5b**) on a combined FA-RA treatment compared to the 13-cis-RA treatment alone. This is possibly due to the role of FA in folate-methylation cycle (Fig. **6**). The 5-methyl-THF, a methyl group donor for folate and methylation cycles is essential for synthesizing S-adenosyl methione (SAM), a universal methyl group donor necessarily for transmethylation reactions (*i.e*. DNA methylation), normal gene expression and morphogenesis [39]. Appropriate temporal and spatial expressions of developmentally regulated genes are maintained by methylation process. This process is absolutely critical to morphogenesis completion and development of normal phenotype. Therefore, inadequate supply of methyl groups could result in changes in DNA methylation pattern, and alteration of gene expression [40-42], and cell differentiation [43, 44].

The underlining mechanism supporting the protective effect of FA on RA induced midfacial process malformation has not yet been investigated. However, adverse effects of RA on methylation and folate cycles of other tissues have been demonstrated in animals and humans. Administration of a large dose of either 13-cis-RA in rats showed an increased number and activity of hepatic glycine N-methyltransferase (GNMT) [45, 46]. The GNMT is a key protein that regulates methyl group metabolism by regulation of SAM levels and SAM/SAH ratio [47] (Fig. **6**). The GNMT catalytically cleaves SAM, thus an up-regulation of GNMT expression leads to the loss of available methyl groups [45, 48], and subsequently impairs transmethylation process. Therefore, hepatic DNA, a substrate for SAM-dependent transmethylation, was hypomethylated after RA treatment [49]. A short-term supplementation (28 days) with 13-cis-RA (0.5 mg/kg/day) in young and elderly healthy adults decreased the plasma level of 5-methyl-THF [50] and consequently perturbed the methylation and folate cycles on the target tissue which may be rescued by supplementation of FA and its metabolic molecules. Furthermore, other studies found that the 5-methyl-THF, a metabolized form of FA, inhibited the GMNT activity induced by RA [51, 52] (Fig. **6**). Since the evidence demonstrated that RA induced the loss of methyl groups, the most putative mechanism of RA-induced malformation of midfacial processes may be caused by impairment of transmethylation process or DNA hypomethylation. As a result, RA may inappropriately impact gene expression in the epithelium and mesenchyme causing the malformed midface. Previous study reported that the ectopic expression of *RARβ* in excess RA-treated embryos was correspondingly observed at the area of facial mesenchyme in LNPs with abundant apoptotic cell death [31]. Taken together, the embryos pre-treated with FA followed by 13-cis-RA achieved a high plasma level of FA and its derivative molecules leading to an increased level of methyl group donors necessary for DNA methylation. Consequently, gene expression, cell proliferation and cell survival were well controlled enabling outgrowth of the midfacial processes of FA-RA treated embryos. In addition, FA and its metabolic molecules are the source of DNA synthesis (Fig. **6**). FA is reduced to tetrahydrofolate (THF), and 5, 10-methylene-THF which then serve as carbon group donors for nucleotide synthesis (purine and pyrimidine) (Fig. **6**). Our results also showed that the action of FA was inhibited by 13-cis-RA (Fig. **5b**). As noted, RA can induce the loss of methyl group donor such as 5-methyl-THF necessary for folate and methylation cycles [50]. To replenish 5-methyl-THF on these cycles, 5, and 10-methylene-THF are utilized causing the lack of substrate for DNA synthesis, perturbing cell proliferation activity as shown by the low numbers of PCNA-positive cells in all midfacial processes of 13-cis-RA treated embryos. Collectively, this study demonstrated the protective effect of FA on 13-cis-RA-induced cellular defect of midfacial processes.

## CONCLUSION

Pretreatment of FA followed by 13-cis-RA in rat embryos significantly decreased the cellular damage of developing midfacial processes. The results showed beneficial effects of FA against the teratogenic effects of 13-cis-RA on this target tissue. Further investigations are needed to clarify the underlying mechanisms of their interactions in order to uncover an applicable prevention of midfacial malformation in infant induced by 13-cis-RA administration during pregnancy.

## Figures and Tables

**Table 1:** The midfacial morphogenesis score for cultured E11.0 rat embryos for 48h assigned by (I) the formation and mergence of midfacial processes and (II) the degree of MNPs merged each other at the midline, then (I) and (II) were summarized to present their midfacial morphogenesis in all treatment groups (adapted form [26]).

(I) Formation and Mergence of Midfacial Processes	Score	(II) The degrees of MNPs Merged each other at the Midline	Score
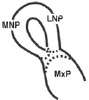	1= MNP, LNP and MxP formed, but not joined and merged	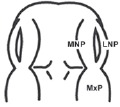	1= MNPs, not joined and merged each other at the midline
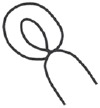	2= MNP, LNP and MxP formed and joined, but not merged	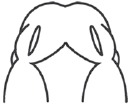	2= MNPs, joined, but not merged each other at the midline
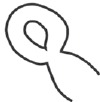	3= MNP, LNP and MxP formed and merged	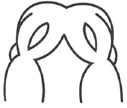	3= MNPs, merged each other at the midline
